# Assessing global Sentinel-2 coverage dynamics and data availability for operational Earth observation (EO) applications using the EO-Compass

**DOI:** 10.1080/17538947.2019.1572799

**Published:** 2019-02-05

**Authors:** Martin Sudmanns, Dirk Tiede, Hannah Augustin, Stefan Lang

**Affiliations:** Department of Geoinformatics, University of Salzburg, Salzburg, Austria

**Keywords:** Sentinel-2, metadata, cloud cover, scene coverage, global analysis, big Earth data, digital earth

## Abstract

Sentinel-2 scenes are increasingly being used in operational Earth observation (EO) applications at regional, continental and global scales, in near-real time applications, and with multi-temporal approaches. On a broader scale, they are therefore one of the most important facilitators of the Digital Earth. However, the data quality and availability are not spatially and temporally homogeneous due to effects related to cloudiness, the position on the Earth or the acquisition plan. The spatio-temporal inhomogeneity of the underlying data may therefore affect any big remote sensing analysis and is important to consider. This study presents an assessment of the metadata for all accessible Sentinel-2 Level-1C scenes acquired in 2017, enabling the spatio-temporal coverage and availability to be quantified, including scene availability and cloudiness. Spatial exploratory analysis of the global, multi-temporal metadata also reveals that higher acquisition frequencies do not necessarily yield more cloud-free scenes and exposes metadata quality issues, e.g. systematically incorrect cloud cover estimation in high, non-vegetated altitudes. The continuously updated datasets and analysis results are accessible as a Web application called EO-Compass. It contributes to a better understanding and selection of Sentinel-2 scenes, and improves the planning and interpretation of remote sensing analyses.

## 1. Introduction and motivation

Each of the Copernicus Sentinel-2A and -2B satellites and downstream processing chains generate 1.7 terabytes of Level-1C (L1C) scenes per day, with the aim to provide regular global coverage (ESA 2017b, 14). Due to the European Copernicus programme’s free, full and open data access policy, the whole archive, currently consisting of millions of scenes, is available to everyone (Breger 2017; European Commission 2013). Financial constraints in purchasing high-resolution data are no longer an obstacle for analysing a massive amount of scenes. The data is increasingly being used by the community to produce information on a global or continental scale, e.g. with tools and methods for managing and processing the unprecedented data volume (Baumann et al. 2016; Purss et al. 2015; Gorelick et al. 2017). Many natural phenomena as well as human-Earth interactions are inherently broad in the spatial extent, multi-scale, and trans-border. The environmental domain especially benefits from increased spatial and temporal resolutions as well as the ever-growing processing power, allowing investigation of increased study area sizes using high-resolution (HR) imagery. Recent and future analyses might not use a single satellite image or only a fraction of the archive, but instead potentially hundreds or thousands of scenes, or even the entire archive and therefore are one of the most important facilitators for the Digital Earth. Examples for extracting global or continental ecosystem dynamics from remote sensing images include surface water (Pekel et al. 2016; Mueller et al. 2016) and forests (Hansen et al. 2013; Gill et al. 2017).

Both the technical specification of the satellites, such as spatial resolution, swath size and revisit times (Drusch et al. 2012), and the technical access to data seems to be relatively straightforward. The latter has been dramatically simplified thanks to platforms such as the Copernicus Open Access Hub, Google Earth Engine (GEE) (Gorelick et al. 2017), Amazon Web Services (Amazon 2016), national initiatives like the Austrian Earth Observation Data Centre (EODC) (EODC 2018b), national ground segments and the Copernicus Data and Information Access Service (DIAS) (Copernicus 2017). Based on the growing Earth observation (EO) domain and simplified data access, ideas are increasingly incubated into business applications that provide, as well as require, reliable data availability or operational services. Technological advances on data access might lead to the wrongful conclusion that the underlying data coverage and data quality are homogeneous, and every algorithm works well on any geographic location. There are numerous projects, start-ups and companies that could benefit from explicit and systematically acquired knowledge about whether their approach is feasible, given the temporal coverage and cloud cover patterns. Example applications include the detection of crop status and optimal harvesting time, flood management and assessing water quality during the summer.

The spatio-temporal characteristics of scenes in a time-series analysis or in large study areas are complex. Differing cloudiness across the Earth, varying acquisition frequencies at different geographic locations, overlapping acquisitions and the acquisition plan all affect the data availability and quality. For example, a time-series analysis with a study area in Europe uses images with different average cloud cover or acquisition frequency than the same method applied for analysis in tropical regions or deserts in Australia. Analysts who intend to study areas larger than a single scene or consider to use multiple observations in time, have to take the inhomogeneity of the underlying data into account before designing the algorithm and interpreting the results.

Considering the inhomogeneity of data and its spatio-temporal characteristics in analysis over vast areas is not a simple task. Tools for analysis and visualisation of global data characteristics and quality have not been developed as quickly as technical access and storage. Currently, users can get information of global data distribution using a Sentinel-2 coverage map of the scenes available for example at EODC (EODC 2018a), or a coverage visualisation as heat map of the data distribution published by ESA in the Sentinel Data Access Annual Report (Serco 2017, 22). The company Mundialis maintains a Web application for searching Sentinel-2 scenes and provides a coverage visualisation as heat map as an optional background layer (Mundialis 2018). The Sentinel-2 coverage can be viewed in the ESA Copernicus Sentinel App. Still, none of the maps or visualisations are machine-readable, available for more than a single, fixed period of time, nor provide sufficient detail for reliable, in-depth interpretation.

Systematic and spatially explicit aggregated information about the coverage, availability, or cloud cover of Sentinel-2 scenes is currently not accessible to the public. Since Sentinel-2A has been operational for almost three years now, we think that an assessment regarding the spatio-temporal coverage and the availability of scenes is justifiable. Several studies in this respect were published for the Landsat archive. Wulder et al. (2016) report on the status of the archive, including spatial and temporal distribution of the number of available scenes. Ju and Roy (2008) and Kovalskyy and Roy (2013) report on the cloud statistics of the Landsat scenes. To the best of the authors’ knowledge, a systematic analysis of multiple variables of global Sentinel-2 metadata has not yet been conducted or made publicly available.

The global spatio-temporal dynamics represent the varying spatial coverage and data quality, e.g. cloud cover, as well as temporal changes, e.g. through seasonal shifts. Knowing the overall distribution of Sentinel-2 scenes as well as its inherent spatio-temporal dynamics is essential to accurately estimate the risks and reliability of a remote sensing project already in the planning phase, developing transferable methods, assessing the method’s transferability and interpreting the result of analysis. For example, does an algorithm, which works well on data from study area ‘A’, also work on study area ‘B’, given the influential factors on the data level, such as the number of acquired scenes in a certain time period or the average cloud cover? Aside from the technical and economic dimensions, this knowledge also has scientific and didactic dimensions. Curating and communicating the spatio-temporal coverage dynamics and availability might be used for Sentinel-2 outreach and education, e.g. to show how massive the data volume really is, which areas are covered, or why there are few scenes in the Antarctic/Arctic regions during the respective winters.

A systematic, near real-time analysis of global Sentinel-2 scenes based on their metadata produces information on the inhomogeneity and spatio-temporal dynamics of remote sensing data. Metadata are limited to some image wide statistics, but have less data volume, are easier to understand and allow for interactive real-time queries and geovisualisation. Therefore, the presented approach is to harvest and analyse global Sentinel-2 metadata, and make it accessible to the public in a Web-based tool, called EO-Compass. This tool can be applied as an upstream meta-tool for big data technology initiatives, analyses on a continental or global scale, and more broadly in all areas located in the Sentinel-2 HR domain. For example, a user who is interested in monitoring deforestation in Indonesia using a time-series of scenes will be able to access relevant information, including the observation frequency, historical information about the cloud coverage, or the average time between cloud-free acquisitions, with only a few interactions that have quick response times.

This paper reports on results from an analysis of the availability of scenes, average cloud cover, cloudiness and availability of scenes with respect to cloud cover, and introduces the EO-Compass as a new tool for the EO community. The remainder of the paper revisits the Sentinel-2 mission, including its context in the European Copernicus programme, characteristics and metadata expected from the acquisition. Further, it describes EO-Compass and its individual components. The results are shown and discussed to illustrate the aggregated metadata statistics, and the future outlook of the project is presented.

## 2. Data and methods

### 2.1. The Sentinel-2 satellites

The Sentinel-2 mission is part of the European Copernicus EO Programme (Drusch et al. 2012). The mission operates two satellites, Sentinel-2A (launched on: 23 June 2015) and Sentinel-2B (launched on: 7 March 2017) (ESA 2017c). In addition, Sentinel-2C and -2D are planned, but will be launched when 2A or 2B reach their end of life. Two satellites always form a constellation in orbit, which are expected to collect several terabytes of data per day over the next years. The equatorial repeat cycle of each Sentinel-2 satellite is 10 days, which, when combined with each other, averages to be five days and increases in frequency at higher latitudes (Li and Roy 2017). The orbit is sun-synchronous at 786 km altitude with a 10:30 a.m. local time descending mode; a compromise between minimising cloud cover, ensuring suitable sun illumination, and similar acquisition situations to other satellites, e.g. Landsat (Gascon et al. 2017). The Multi Spectral Instrument (MSI) on both satellites is a high-resolution multispectral imager with 13 spectral bands combining different spatial resolutions and a swath width of 290 km. Typical application fields include monitoring land surface and climate change, detecting land cover/land use and changes, and supporting disaster relief and emergency management. The Level-1C (L1C) scenes provided to the users are composed of 100×100 km tiles, called granules, delivered in UTM/WGS84 projection (ESA 2015) as ortho-images using a digital elevation model (DEM). The constant ground sampling distance (GSD) is 10, 20 and 60 m depending on the native resolution of the different spectral bands.

The Sentinel-2 mission aims to achieve evenly spread global coverage. According to the Sentinel High Level Observation Plan, the baseline observation scenario is (ESA 2017b, 9)

to cover all land surfaces (starting 20 km from the coastlines) between 56° South latitude (Cape Horn in South America) and 84° North latitude (north of Greenland) including major islands, EU islands and all the other small islands located at less than 20 km from the coastline, the whole Mediterranean Sea as well as all inland water bodies and closed seas. (9)

Additional calibration sites (e.g. in Antarctica) are also covered. The actual Sentinel-2 observations in full operation mode are strongly related to these requirements but will be defined in more detail based on further requirements, such as repetitiveness, seasonal variations and specific priorities (ESA 2017c).

### 2.2. The Sentinel-2 scene metadata

The term scene refers here to a multispectral image. The scene(s) are packaged together with additional data (e.g. cloud masks) into products. The metadata of each L1C product entry contains product specific information, including: product title; filename; format; download link; universally unique identifier (UUID); cloud cover percentage (as single value for the whole image footprint); sensing start and end timestamps; archive ingestion timestamp; the product’s footprint in both geography markup language (GML) and well-known-text (WKT); absolute and relative orbit numbers; orbit direction (e.g. ascending or descending); processing level (e.g. Level-1C); processing baseline (e.g. 02.06); product type (e.g. S2MSI1C for Sentinel-2 multispectral instrument Level-1C); scene file size; and unique UTM granule name (e.g. 33TUN), which is only consistently accurate for products containing a single granule. Since the 27th of September 2016, ESA publishes the scenes as individual UTM tiles (ESA 2016) with the granule name in the metadata (Serco 2017, 13). Scenes that were published before do not have this information located in a single metadata file, rather distributed throughout the product folder. The L1C products also include simple cloud masks, distinguishing between opaque and cirrus clouds based on radiometric thresholds on bands 1 (433–453 nm) and 12 (2100–2280 nm; opaque clouds), while cirrus clouds are detected with band 10 (1365–1395 nm) (Gascon et al. 2017). Although the exact algorithm and the individual thresholds are not publicly accessible, the average cloud cover of the scenes provided as scalar value in the L1C metadata was used in this study. These values are highly relevant as they are used in the filter conditions by users of the Copernicus data distributors, either through the graphical Web application or the API (Application Programming Interface).

### 2.3. Methods

The metadata for all available Sentinel-2A and 2B L1C scenes have been downloaded from the Copernicus Open Access Hub (https://scihub.copernicus.eu/) into a PostgreSQL database and are regularly updated every few days. A search and download software for Sentinel-2 scenes, which was developed in-house was modified for downloading only the metadata and automatically inserts them into the database.

Since there were changes in the data packaging in 2016, the scenes from 2017 have been selected for the examples shown in this study. However, for the first months of 2017 only scenes from Sentinel-2A are available, since Sentinel-2B was not yet in orbit or in the ramp-up phase. Further, filters were set for differencing between Level-1C and Level-2A scenes and also to differentiate between seasons. Other filters, including platform (2A/2B) and processing baseline (e.g. 2.04–2.06) were not set, such that the first dataset contains the metadata from scenes acquired in 2017 by Sentinel-2A or Sentinel-2B and being processed to Level-1C. A second dataset contains the metadata of all scenes processed to Level-2A in 2017. An overview over the number of scenes used in this study with different filters is provided in Table 1.

**Table 1.** Overview over the number of scenes used in this study. Different filters reflect combinations between the satellite and the processing level.

Filter	Number of scenes
Level-1C total	1.359.070
Level-2A total	77.657
Sentinel-2A total	1.401.502
Sentinel-2B total	35.225
Sentinel-2A Level-1C	1.326.852
Sentinel-2B Level-1C	32.218
Sentinel-2A Level-2A	74.650
Sentinel-2B Level-2A	3.007

Using all available Level-1C scenes from both Sentinel-2A and Sentinel-2B, four variables were queried from the database and spatially grouped by granule footprint: number of scenes, average cloud cover, cloudiness and availability. The number of scenes was calculated by counting the available products. The average cloud cover was derived by calculating the average for each granule footprint over time. In the study, the cloudiness is defined as the ratio between cloud-free scenes and cloudy scenes, with cloudy scenes having more than 10% cloud cover. In other words, it describes the relative amount of cloud-free scenes for each granule footprint. Further, the average duration between two cloud-free acquisitions is called availability in this study. Not considered in this study was the processing duration, i.e. the time difference between acquisition and publication in the archive, because of possible distortions during the ramp-up phase of Sentinel-2B and ongoing reprocessing activities by ESA. Although a short comparison between the coverage of Level-1C and Level-2A data is provided, Level-2A data were not systematically considered, because they are at present only partially available within a limited geographical and temporal range.

The geons concept (Lang et al. 2014) is used to delineate regions showing homogeneity of the two dimensions data coverage and average cloud cover. The result can be described as regions of different suitability. Therefore, the number of scenes and average cloud cover were converted into a raster representation where both variables are represented as individual bands. The values of each band were normalised to values from 0 to 255 individually. The cloud cover values were inverted, such that high values represent low amount of cloud cover. This creates a consistent representation of ‘better’ conditions, e.g. where high values represent more scenes as well as low average cloud cover. The artificially generated image was segmented using the multiresolution segmentation algorithm (Baatz and Schäpe 2000) with user specified parameters (Scale parameter: 200, Shape: 0.1; Compactness: 0.1; Layer weighting: 1,1) and classified (noData: Mean number of scenes < = 1; ‘Very High’: Mean cloud cover (c) > 195 && Mean number of scenes (n) > 18 && unclassified; ‘High’: c > 165 && n > 15 && unclassified; ‘Medium’: c > 115 && n > 15 && unclassified; ‘Low’: c > 75 && n > 15 && unclassified; ‘Very Low’: (c > = 75 || n < = 15) && unclassified) in the eCognition software (Trimble Geospatial).

These types of queries can be reproduced and executed interactively in the EO-Compass’ Web frontend, i.e. with a response time of a few seconds for a global analysis. A short introduction of the EO-Compass software is given in the next section, but the focus of this paper is on the report of the results of the exploratory analysis rather than the technical implementation.

## 3. EO-Compass

### 3.1. Overview

The EO-Compass is a tool, developed and designed in-house, to investigate the inherent spatio-temporal dynamics of the Sentinel-2 archive and the performance of the Sentinel-2 satellites in an exploratory manner based on scene metadata. It can serve as a planning tool to examine and compare study areas with respect to their data suitability and characteristics, and is also intended to facilitate detecting long-term changes and trends, e.g. within seasonality of cloud cover. The EO-Compass can assist the dissemination and communication of information about the Sentinel-2 mission and acquired imagery with the aim to improve educating students and informing the public about the mission.

The metadata database can be used for different use cases. Based on the aforementioned variables, different thematic maps can be created and visualised. Since the maps illustrate the values of a variable based on the granule footprint, they are called granule-maps in the EO-Compass. Further, descriptive statistics in an infographics-style per individual granule can be retrieved. Additionally, the status of the Sentinel-2A and -2B satellites (e.g. current position, predicted orbits) are cartographically visualised and overlaid on the current acquisition plan and enriched with weather forecast information.

### 3.2. Database and datasets

The EO-Compass stores the metadata of the scenes in a PostgreSQL database system, which is spatially enabled by the PostGIS extension. Data about the orbit parameters to calculate the current position and to estimate the next orbits are collected from the celestrak website (https://celestrak.com/). Publicly available information about the Sentinel-2 satellites and mission is either collected from the ESA website or provided as an HTTP hyperlink (e.g. acquisition plans, Sentinel-2 news, data quality reports, and mission reports).

### 3.3. User interface

The Web application’s frontend is a graphical access point to the database, implemented using Ember.js and Leaflet. Depending on their geographical properties, the data are either retrieved using a REST (Representational State Transfer) API or OGC (Open Geospatial Consortium) services.

**Figure 1.**
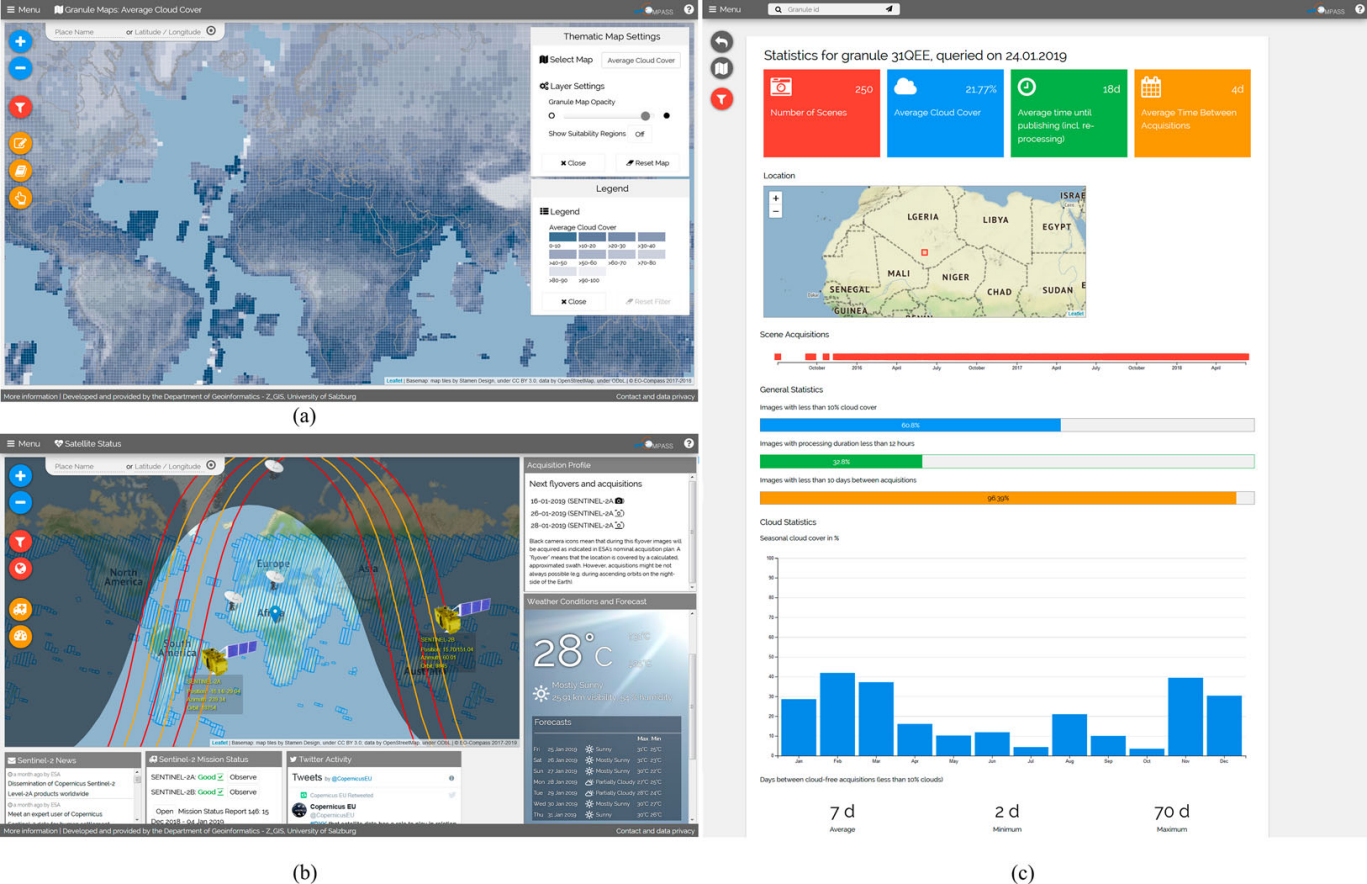
Overview over the EO-Compass. (a) User-selected granule map, showing the percentage of cloud-free scenes (blue – low amount of average cloud cover, white – high amount of average cloud cover). (b) Live positions of the satellites and predicted orbits allow estimating next acquisitions. They are overlaid on the current acquisition plans. (c) An infographic for a single granule reveals the temporal pattern, including the cloud cover seasonality.

Possible user interactions are shown in Figure 1 and include retrieving granule maps, i.e. spatial distribution of selected variables, retrieving location-based temporal statistics, and viewing acquisition prediction. The image footprints of the granule maps can be filled with different variables (e.g. number of scenes), which are calculated at any time needed directly in the database. Due to the amount of UTM tiles (approx. 65,000 polygons), a purely WFS-based (vector) publication was found to be too slow or not feasible, while a WMS-based publication lacked interactivity. Therefore, vector tiles were used here to allow interactivity to a certain degree while still keeping reasonable performance. The EO-Compass is currently installed on a Red Hat Enterprise Linux virtual server with 4 CPU, 16 GB RAM and all-use virtual storage, which yield average response times of a few seconds for generating the maps.

## 4. Results

### 4.1. Global data coverage analysis

**Figure 2.**
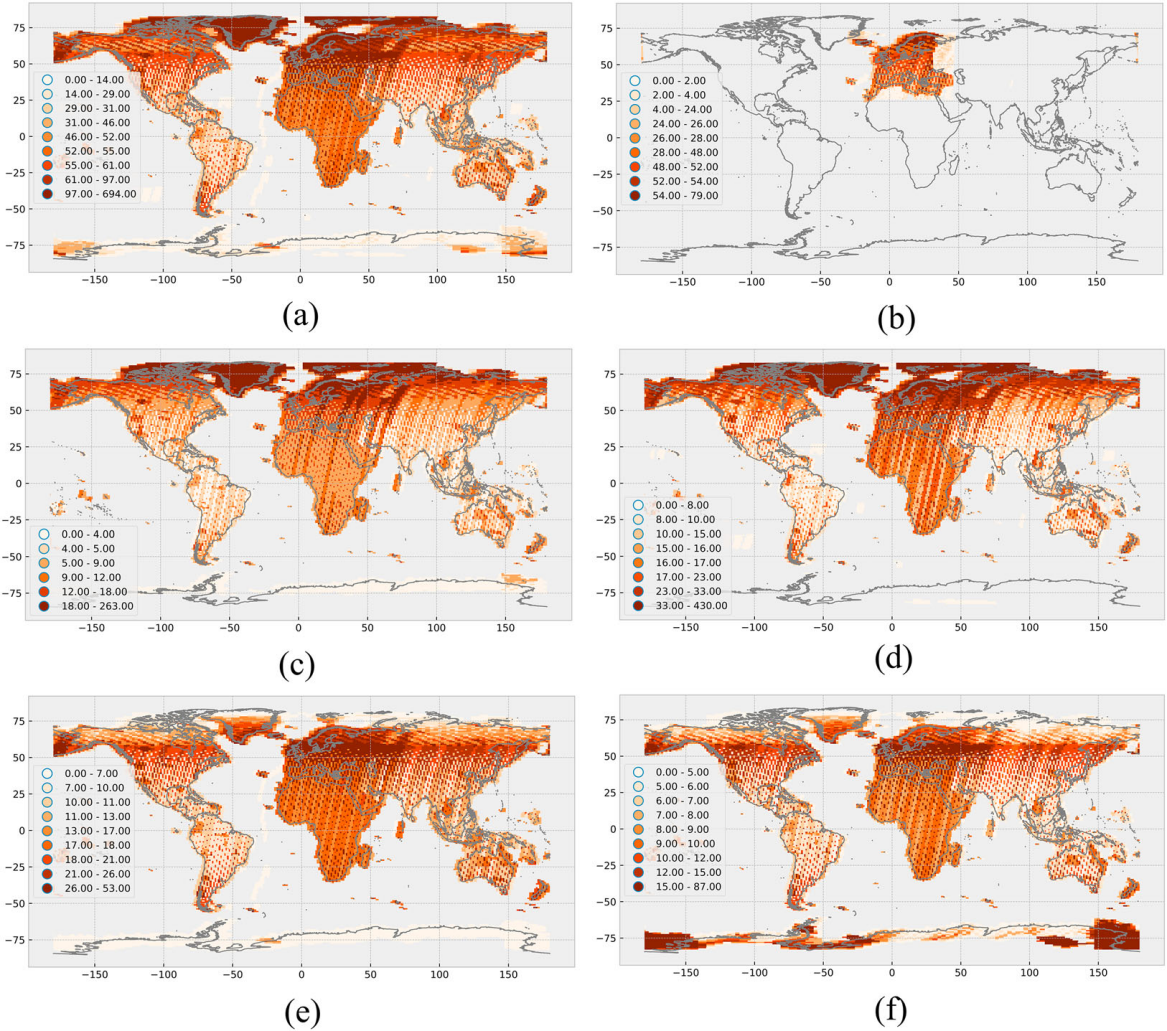
Global spatial distribution of number of Sentinel-2A and 2B scenes per granule, which were captured in the year 2017. (a) Level-1C scenes, (b) Scenes, which have been processed up to Level-2A. (c) Level-1C scenes acquired in spring (21.03.2017–20.06.2017). (d) Level-1C scenes acquired in summer (21.06.2017–21.09.2017). (e) Average cloud cover for autumn (22.09.2017–20.12.2017). (f) Average cloud cover for winter (1.1.2017–20.03.2017 and 21.12.2017–31.12.2017). In all figures the values are classified using quantiles, where the upper boundary is included. The seasons are selected as they are commonly used in the northern hemisphere including Europe with its Copernicus programme. Own cartographic representation.

The number of Sentinel-2 acquisitions for the year 2017 is shown in Figure 2. In general, high acquisition rates are experienced in Europe, Africa and Madagascar, Greenland, Iceland and New Zealand. In addition, many of the world’s main cities (e.g. New York City, Beijing, New Delhi, Buenos Aires) and some additional locations (e.g. Lena Delta, Ross Sea with the Italian Zuchelli, US McMurdo Antarctic Station) are covered more frequently than their surroundings, which is clearly visible as dark orange spots. Major islands are covered with varying frequency. The seasonality affects position of the sun and therefore the illumination, which limits the acquisition in very high latitudes to certain time spans.

### 4.2. Global average cloud cover analysis

**Figure 3.**
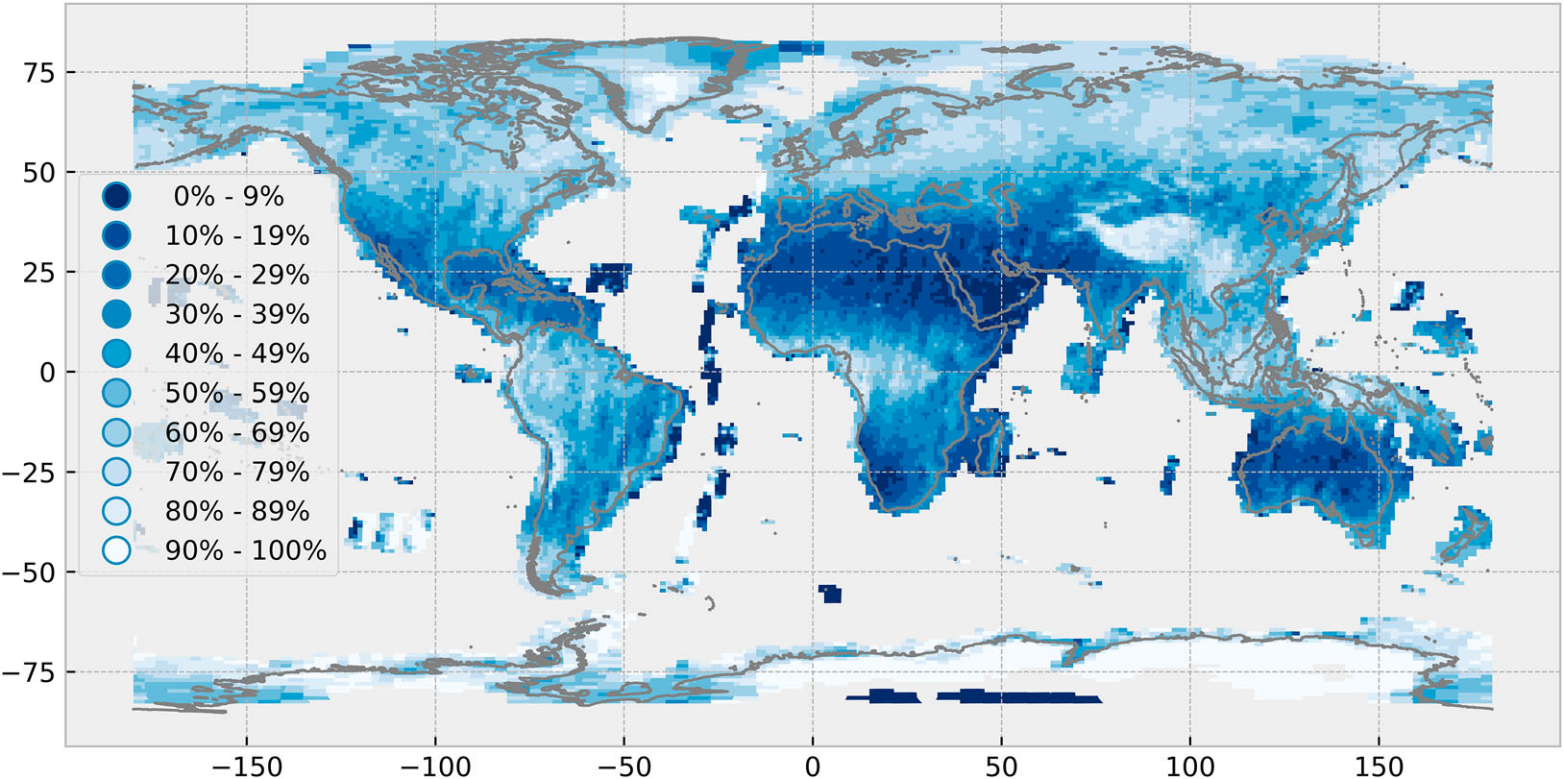
Global spatial distribution of the average cloud cover of Sentinel-2A and Sentinel-2B Level-1C scenes acquired in the year 2017. Own cartographic representation.

Figure 3 illustrates the overall average cloud cover for the year 2017. Regions that are covered by scenes with a high percentage of clouds are the Arctic, central Greenland, the Himalayas, parts of the Andes, and the tropical parts of Indonesia, Africa and South America. Scenes with low average cloud cover are available in Northern Africa, the Near and Middle East, Southern Africa, the majority of Australia and New Zealand, the Western part of the USA and several islands in the Atlantic ocean.

**Figure 4.**
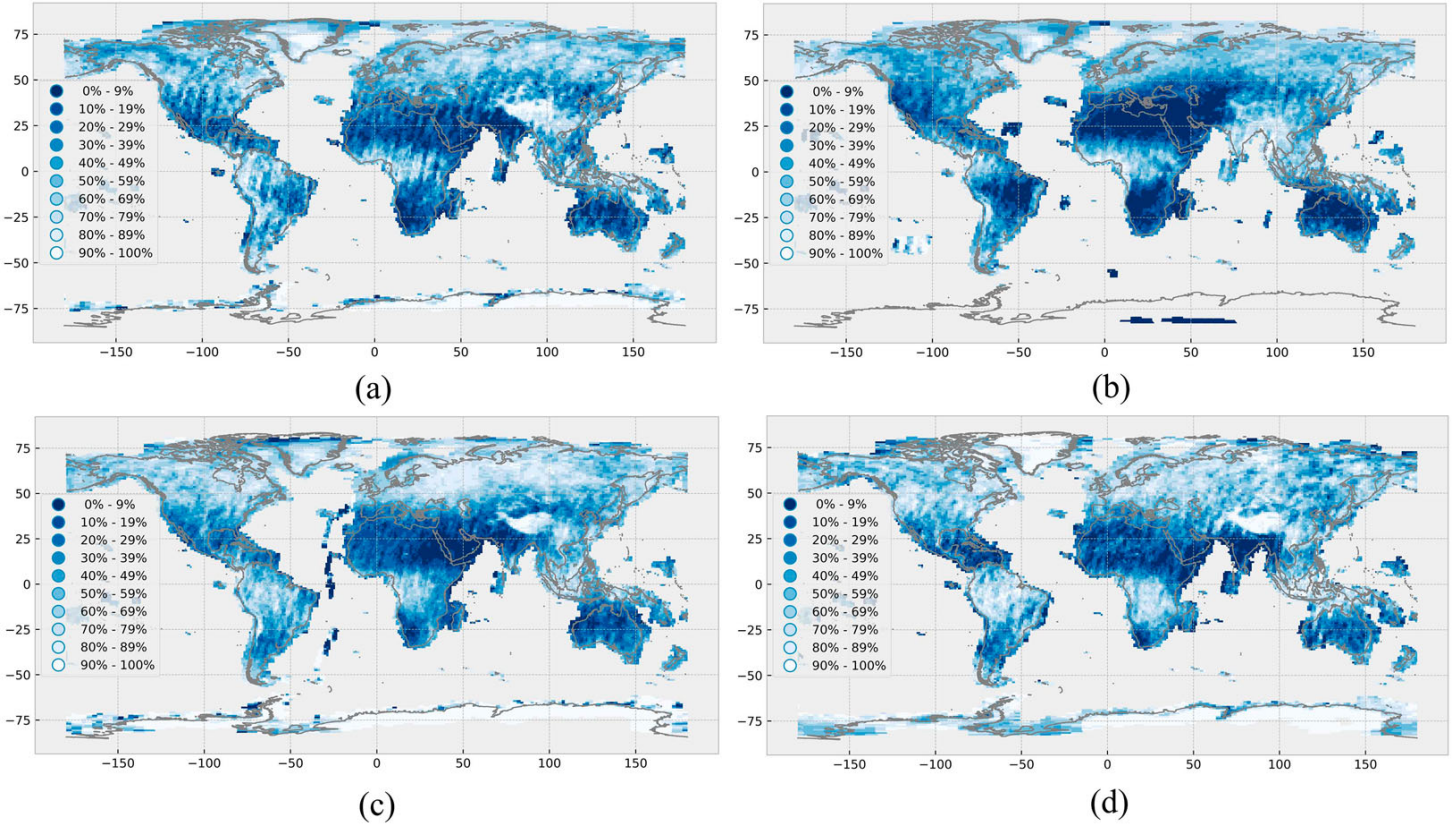
Seasonal shift in cloud coverage, taking a four-seasonal pattern as reference (northern hemisphere as reference) in the year 2017. (a) Average cloud cover for spring (21.03.2017–20.06.2017). (b) Average cloud cover for summer (21.06.2017–21.09.2017). (c) Average cloud cover for autumn (22.09.2017–20.12.2017). (d) Average cloud cover for winter (1.1.2017–20.03.2017 and 21.12.2017–31.12.2017). Own cartographic representation.

Figure 4 illustrates the seasonal variability of the average cloud cover, showing a similar overall pattern compared to Figure 3. However, the seasonal effects are clearly visible. For example, India has high average cloud cover during the summer and low average cloud cover during the winter. Differences between summer and winter are also visible on the African continent, where the location and size of the main region, which is covered by clouds, is oscillating and reflecting the seasonal pattern of the Intertropical Convergence Zone (ITCZ). The average cloud cover over the European continent is more stable and reaches its maximum in autumn.

### 4.3. Global cloudiness analysis

**Figure 5.**
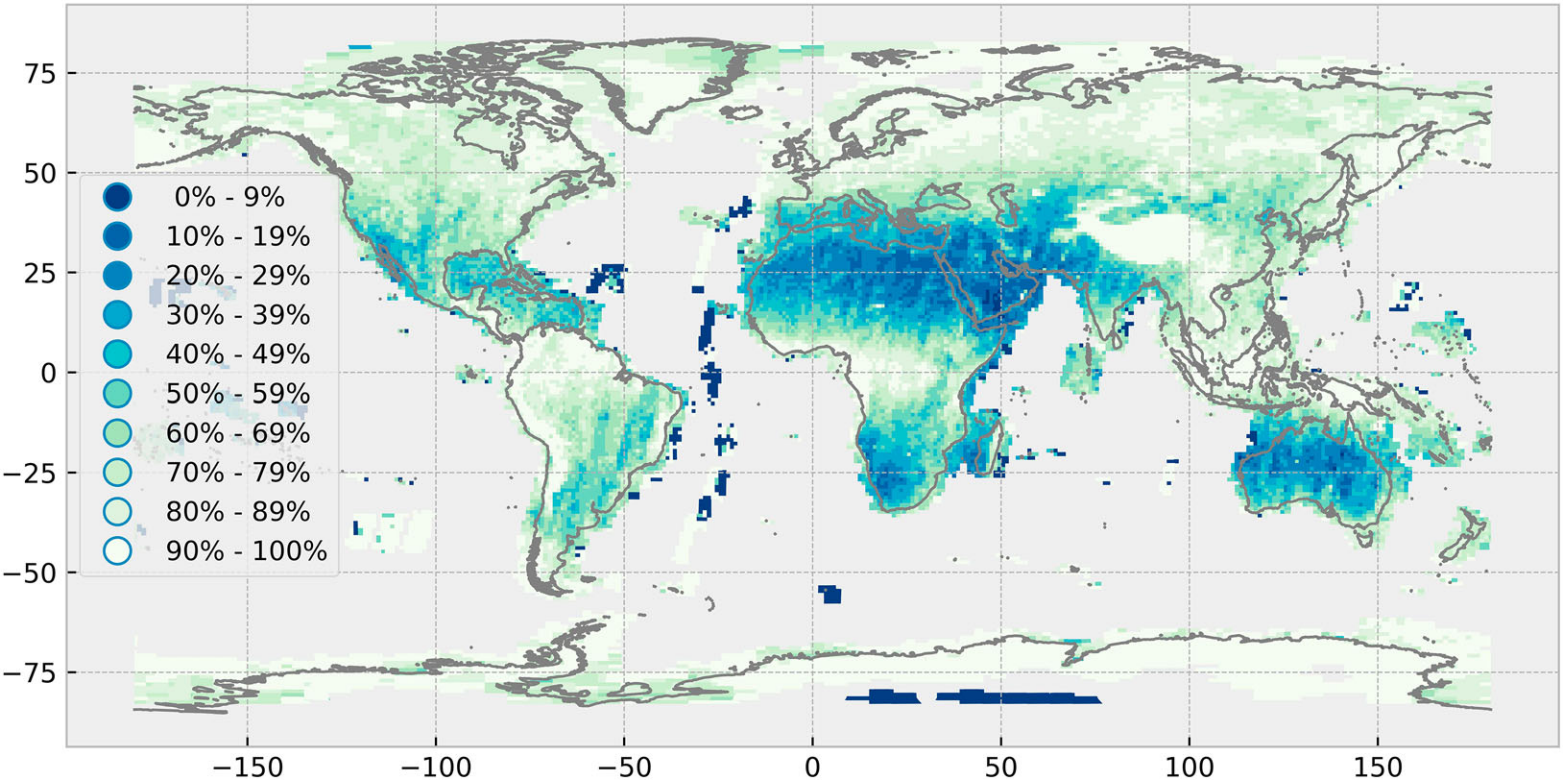
Global cloudiness for 2017. Percentages indicate the fraction of cloud-contaminated Sentinel-2A and 2B Level-1C scenes. 0% means that all scenes are cloud-free and 100% means that all scenes are cloud-contaminated. It does not refer to the amount of clouds within the scenes. Own cartographic representation.

The cloudiness map shows the percentage of cloud-contaminated scenes, with cloud-free being defined as less than ten percent clouds (Figure 5). The number of available scenes influences results to a certain degree, but in general, the same pattern as in Figure 3 can be observed. A low fraction of cloud-contaminated scenes are in the western part of the USA and Mexico, Caribbean, south-eastern parts of South America, northern parts of Africa, the Near and Middle East, parts of India, Southern Africa and Namibia, some parts of Eastern China and the Majority of Australia. In general, large parts of the world have very few cloud-free acquisitions.

### 4.4. Global data availability analysis

**Figure 6.**
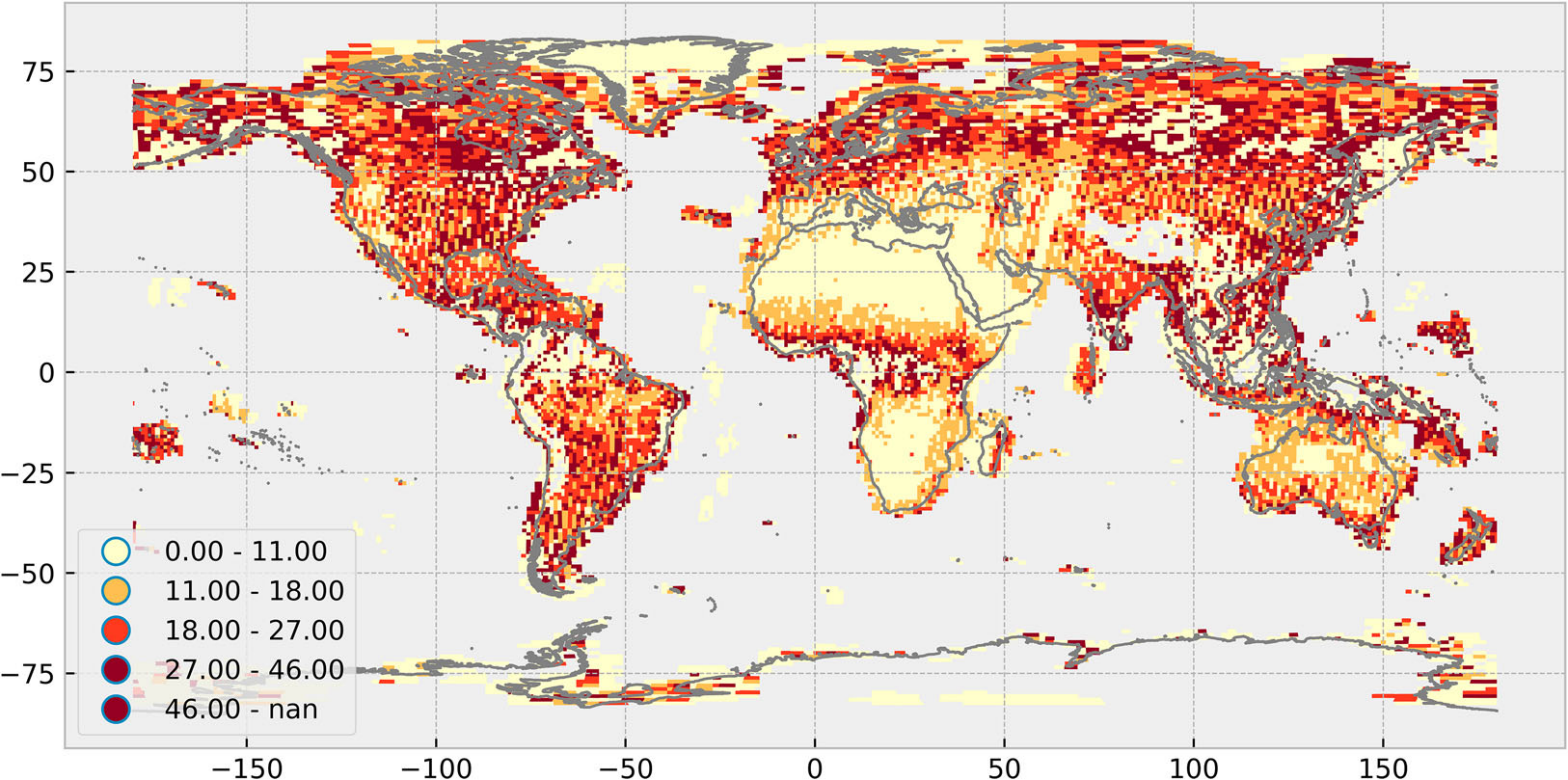
Global data availability of Sentinel-2 Level-1C scenes for 2017. The colours show the average number of days between two cloud-free scenes. The values are classified into quantiles where the upper bounds are included. Interpretations of values for very high latitudes above 75° and below −75° are difficult because few scenes are cloud-free, but multiple acquisitions per day are possible during the polar day and good conditions. Own cartographic representation.

The availability map (Figure 6) shows the average days between two cloud-free acquisitions with cloud-free still defined as less than ten percent of clouds. Following this analysis, data gaps occur in several regions on the Earth including the Andes, Himalayas, Arctic, Antarctic and tropical regions in Africa and Indonesia. The gaps indicate that, for the investigated time frame of 2017, only one or no scenes were cloud-free according to the provided metadata. On the other hand, major parts of Africa, the Near East and some parts of Australia are distinctly visible, where the average duration is less than two weeks. The majority of the passes over these regions can be considered to be cloud-free. Except for the extrema, the average duration is between two and six weeks in Europe, Asia, North and South America, and Australia with New Zealand. In high latitudes, e.g. Antarctic and Arctic regions, many acquisitions are cloud-contaminated. However, due to overlapping orbits and continuous illumination during the polar day, there are occurrences where multiple cloud-free acquisitions are possible in one day with good conditions. The interpretations for these regions are therefore difficult and conclusion should be drawn with care.

### 4.5. Regions of different suitability

**Figure 7.**
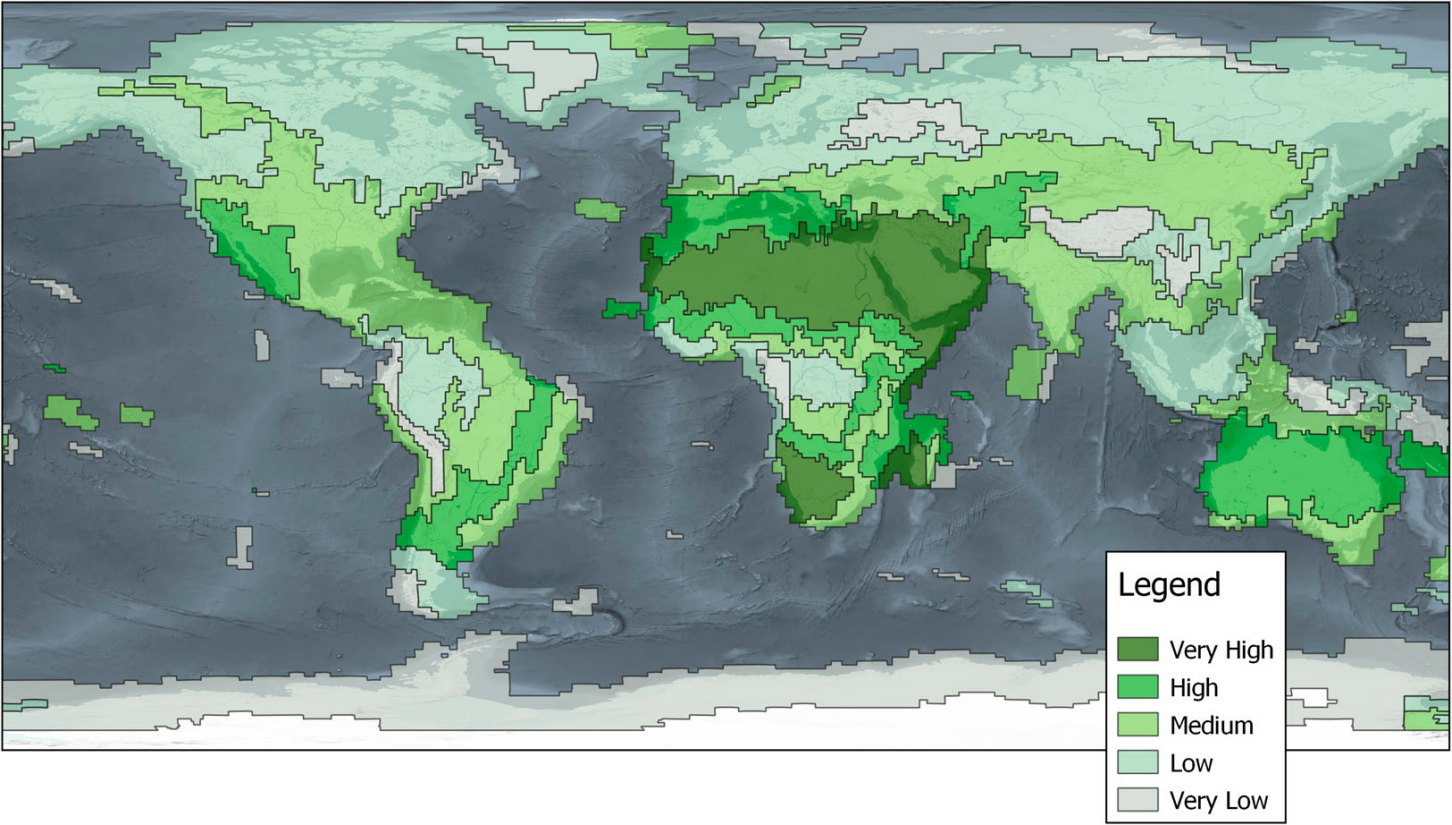
Spatially explicit regions of different suitability for Sentinel-2A and 2B Level-1C data. High suitability means a combination of high coverage and low average cloud cover. The suitability of data at a specific location is decreasing with either a decrease of number of scenes or an increase of average cloud cover. Own cartographic representation; terrain basemap provided by EOX GmbH.

The previous sections showed that the data coverage and average cloud cover are varying in space and time, while the calculated suitability regions in Figure 7 provide a spatially explicit categorisation. Very high suitability can only be reached if the data coverage is high and cloud cover is low, in the case of the scenes acquired in 2017, this was achieved in arid areas in Africa. In other arid areas as well as subtropical areas in Africa, central Asia, Australia and New Zealand as well as North and South America high or medium suitability can be reached. In tropical regions at the ITCZ across the equator as well as regions in higher latitudes towards the North Pole and the South Pole, the high cloud cover is a persistent problem. Some exceptions exist, e.g. in India where the seasonality between high cloud coverage during summer and low cloud cover during winter is not reflected, but averaged out. Specific climate conditions are also carved out in the regions, e.g. the coast of Newfoundland or the southern part of Australia with more tempered climate zones.

## 5. Discussion

The metadata analysis is based on a replicated database to allow sophisticated temporal and spatial queries, which was populated by means of downloading the metadata of all Sentinel-2 scenes. Only scenes that are published in the Copernicus Open Access Hub were considered in this study. Similarly, the analysis did not correct for other changes, if any, in the processing of the L1C scenes by ESA and does not consider pre-operational scenes from the ramp-up phase, which are still being re-processed and added.

The granule map showing the number of scenes reflects the geographical observation scenario as specified in the High Level Operations Plan (ESA 2017b, 9), except for additional observations in Antarctica, which are most likely observations over specific calibration sites. However, the revisit frequency, especially of the mainland of Europe, Africa and Greenland, is twice as high as the rest of the world (ESA 2017d), resulting in a remarkable varying number of scenes. According to Serco (2017, 22), during the ramp-up phase particular emphasis was focused on European and African land masses, but the overall high differences in coverage are likely due to refined acquisition plans with certain priorities and constraints in data acquisition.

**Figure 8.**
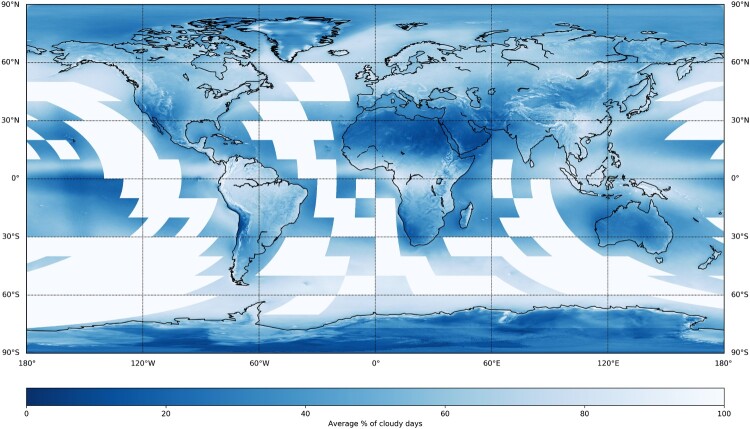
15 years mean annual cloudy days based on data from MODIS. White, quadrangular patches over the oceans indicate missing data. Own cartographic representation with a dataset from Wilson and Jetz (2016). For cartographic representation the dataset has undergone cubic down-sampling into a 0.1° × 0.1° grid.

The average cloud cover generally corresponds with the climate zones and their known cloud occurrences, e.g. ITCZ in tropical regions. Figure 8 illustrates the mean annual cloudy days between 2000 and 2014, based on data by Wilson and Jetz (2016). While the years are different, it aims to serve as a simple visual comparison to the average cloud cover map of 2017 (Figure 3) and shows large agreement. However, there are larger exceptions for the Antarctic and Arctic regions and specific high altitude regions in the Himalayas and Andes.

**Figure 9.**
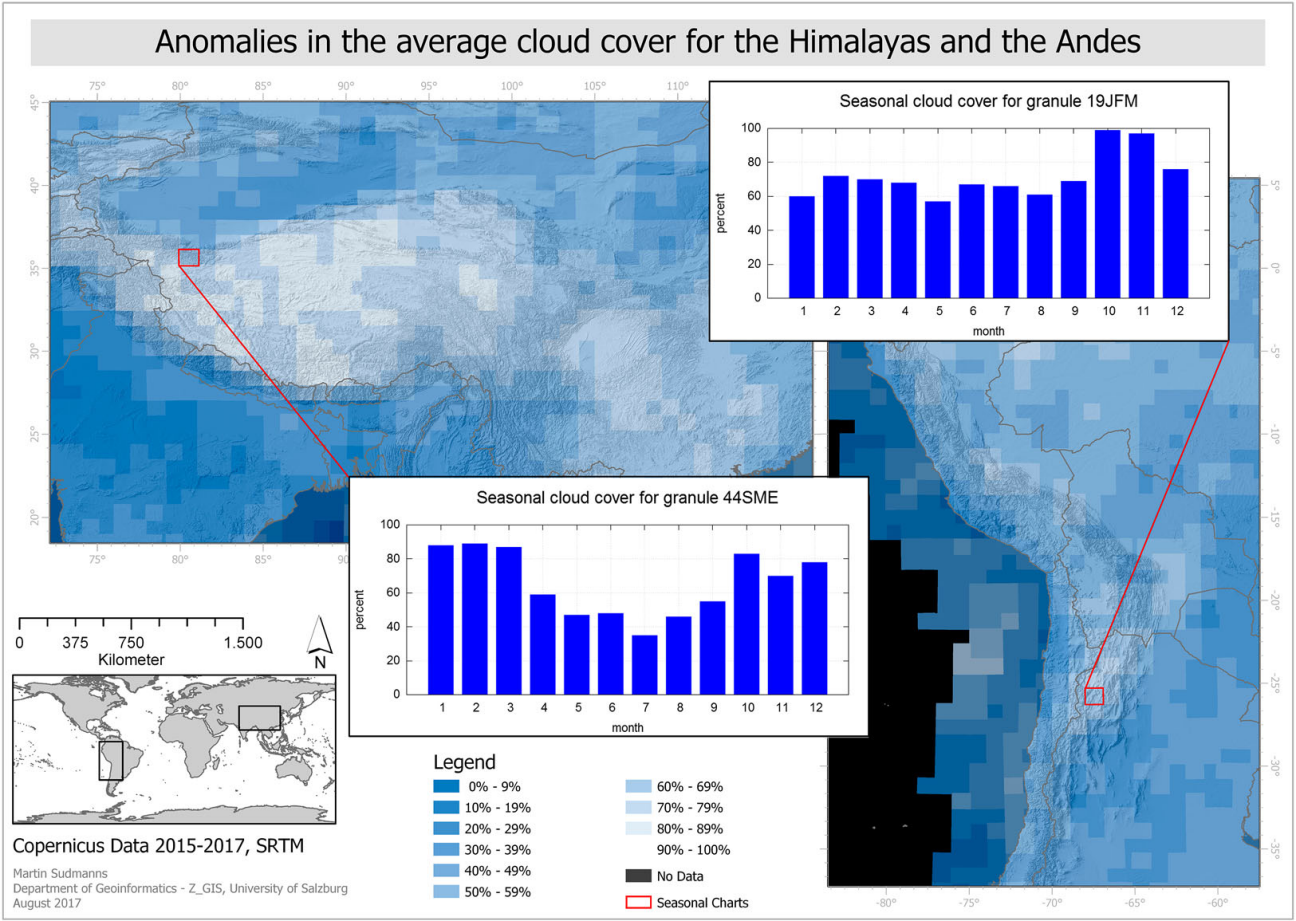
Anomalies in the cloud cover estimation in the high altitudes of the Himalayas and the Andes, revealed by the long-term metadata statistics provided by the EO-Compass. Own cartographic representation.

It seems that ESA’s cloud detection algorithm, based on radiometric thresholds on bands 1 and 12 for opaque clouds and band 10 for cirrus clouds (Gascon et al. 2017), does not work properly in some regions, including those, which are low vegetated and at high altitude (Coluzzi et al. 2018) (Figure 9).

**Figure 10.**
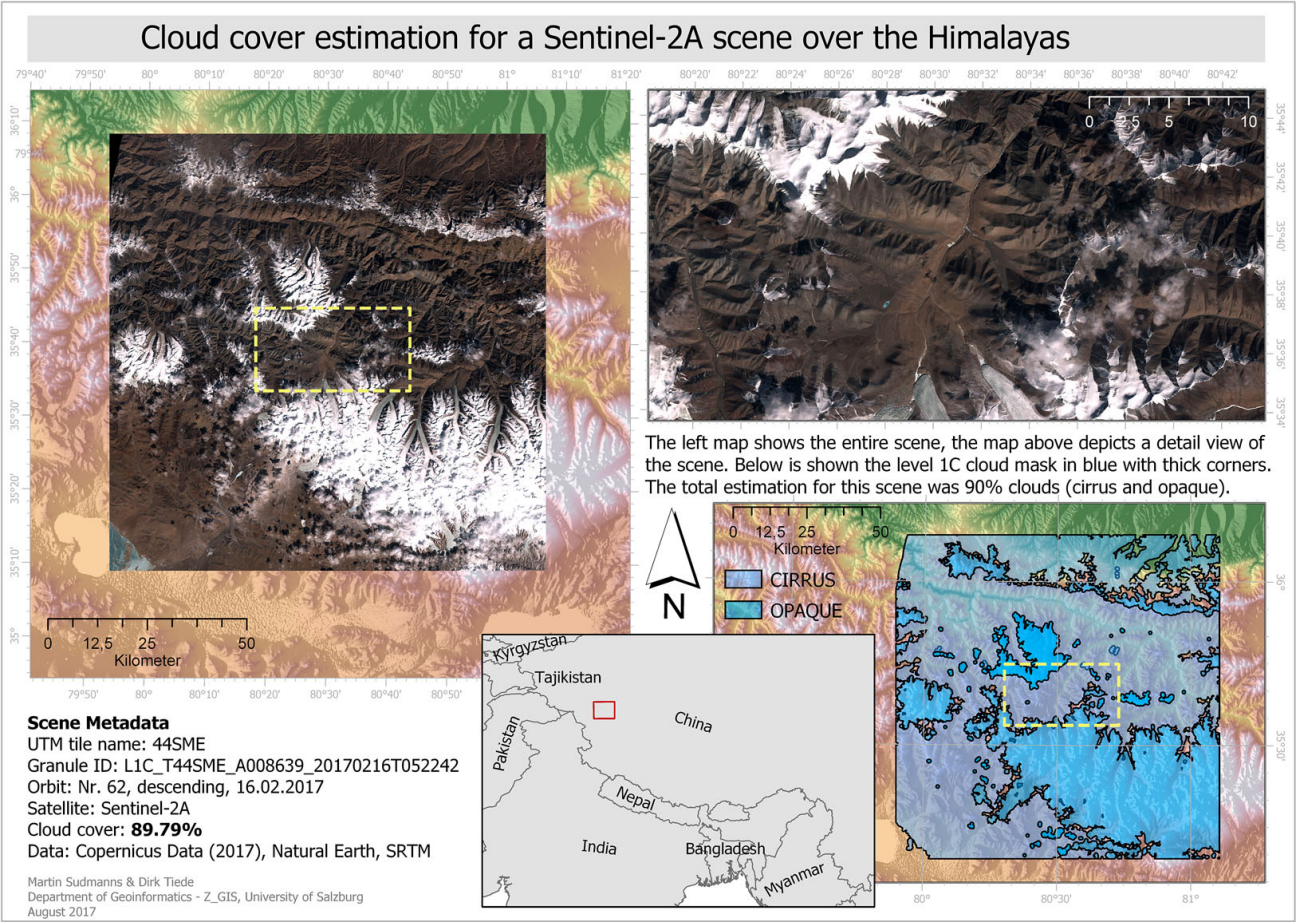
The estimated cloud cover in the metadata for the selected scene in the Himalayas is 90% (see the metadata related cloud mask delivered as part of the product), although a visual inspection shows that the majority of the scene is cloud-free. Own cartographic representation.

A possible explanation might be that the threshold of band 10 is also sensitive to bare soil in non-vegetated high altitudes, yielding incorrect cloud cover estimations, and that snow covered areas are partly interpreted as opaque clouds. Coluzzi et al. (2018) argue that the overdetection errors in high altitudes are due to dry atmospheric conditions. Figure 10 shows an example for a selected Sentinel-2A scene in the Himalayas where almost no cloud cover is present, but the scenes’ attached cloud masks indicate an estimated cloud cover of almost 90%. Such strong outliers can be found mainly in samples from regions in very high altitudes (e.g. Himalayas, Andes) and not in samples from non-vegetated areas in lower altitudes (e.g. deserts). A future improvement of the cloud detection algorithm used by ESA may therefore include a threshold-calibration based on elevation data or a solution which is more reliable in these regions.

Other obstructions in the cloud and cirrus detection, which could affect analyses, are not simple to detect. Examples of these obstructions are haze, thin cirrus, and cloud shadow. If they cannot be removed in the pre-processing for image analysis by methods for atmospheric corrections they might lead – together with other sources of errors – to wrong classification results (Zhu and Woodcock 2012).

The cloudiness and availability maps are an important information source to plan and estimate the reliability of time-series analyses and change detection if specific periods of time need to be covered, as they show the expected time between two cloud-free acquisitions. This can also be of help in the planning process to complement specific areas with acquisitions from other sensors like Landsat. Not surprisingly, the spatial pattern resembles the average cloud cover. However, it also reveals that despite the higher acquisition frequency in Europe, it does not necessarily yield more frequent or successive cloud-free acquisitions.

The regions of different suitability can serve as an integrated spatially explicit indication of a general-purpose suitability of the data, since the coverage as well as the average cloud cover are incorporated. The geon concept applied here is therefore a method to formalise the spatio-temporal suitability, showing an easier way to grasp the dynamics and characteristics of the two selected suitability parameters. In this study, a fixed spatial and temporal reference frame was selected. It can be expected that the delineation may differ by using scene metadata from other/many years. Further, it was calculated for general-purpose and not for application scenarios with specific requirements. Still, it compresses the two data quality variables of one million scenes into a single map, which makes it a helpful tool as a first contact point to evaluate Sentinel-2 data suitability for an operational EO project.

## 6. Conclusion and outlook

Detailed information about the inhomogeneity of data availability and cloud cover as one indicator of data quality is crucial for the success, credibility, interpretation and validation of regional, continental or global analysis. In the context of increased applications using free and open data on a global scale, knowing the inherent spatio-temporal dynamics of the underlying data is essential to successful analysis and interpretation. The presented systematic collection and spatial analysis of Sentinel-2 metadata produce the required information based on the real acquisitions apart from theoretical observation plans.

By means of the EO-Compass, reliable statistics are generated via intelligent spatial metadata analysis as a unique solution for aggregated scene coverage, availability, and quality statistics. It allows users to more intelligently use Sentinel-2 data for better planning and estimation of suitability for multiple use cases and application. It provides spatially explicit statistics, i.e. per granule, and takes varying revisit times into account. Use cases are time-series analysis, cloud-free mosaic or composite production, or in general any analysis, which uses study areas larger than a single granule. The global analysis was also useful for revealing the anomalies in the cloud cover estimation in low-vegetated high altitudes. The remote sensing community is encouraged to use the results and the EO-Compass to be better positioned in the planning, algorithm design and interpretation phase of their projects.

As expected, the percentage of cloud-free scenes in the Sentinel-2 archive is dependent on the geographic location, elevation and seasonality. Still, this study cannot replace a detailed investigation of the individual scenes, as some atmospheric effects might not be adequately represented in the metadata. For example, the work on assessing the detection quality of thin cloud shadow, cirrus and haze is in the early stages (Coluzzi et al. 2018). Further, it could be confirmed that the cloud detection provided with the Sentinel-2 L1C product needs some improvement, in particular a better distinction between snow or bright rock and clouds at higher altitudes. Users of the different data hubs should be aware that in these areas the cloud cover estimation is currently not reliable and should be used carefully as selection criteria in the data search. The data coverage and frequency of sensing is dependent on latitude and the acquisition plans. Europe, Greenland, Africa and important cities were observed more often than other regions of the world. However, the coverage maps alone are not expressive enough for some practical applications. The coverage maps need to be considered side by side with cloud cover statistics. Indicators for the applicability and usefulness of scenes include the proportion of cloud-free acquisitions and the availability as the average duration between two consecutive cloud-free acquisitions.

Investigations of anomalies, which are revealed by the proposed global and systematic analysis of metadata and not published in the current data quality report (ESA 2017a), are difficult. They might require reverse engineering of parts of the L1C product generation processes, e.g. to explain possible reasons for incorrect cloud estimations in non-vegetated high-altitudes. One could argue that, in the current situation, the data is not open in a stricter sense, but the European Commission’s free, full and open policy refers to data access and therefore not necessarily to the data itself (European Commission 2013). If the ‘openness’ would be expanded to a degree that encompasses the processing pipeline alike, it will be easier to investigate these and similar issues. Similarly, the community’s motivation should certainly be to contribute and share their findings and possible improvements alike with the aim of improving the data and processes for everyone and help to deliver the best products possible.

The EO-Compass is under continued development, with planned incorporation of additional features, functionalities and other sensors. Further work will include more variables in the analysis, e.g. processing duration as an indictor for suitability of real-time analysis. Furthermore, the EO-Compass will be expanded with new features helping EO data users to make better decisions. The features are statistics for multi-granule regions, acquisition monitoring with an alarm (e.g. for new cloud-free acquisitions for user selected areas) or multi-satellite querying (e.g. querying constellations when Sentinel 2 and Landsat 8 acquire an image of the same location at the same time). Additional data will be included: basemaps for land use / land cover; the current weather situation; or data about crises and natural disasters. The use of these data enables enrichment of the predicted orbit paths with predicted cloud cover as well as geographic phenomena, which can be translated into real-time information of interest to the public (e.g. ‘Sentinel-2A is now observing forest areas in Siberia, Russia’, ‘Sentinel-2B is currently collecting data about the living environment of 300 million people’, ‘Sentinel-2A is collecting data about the flood that happened in Bangladesh’). A feasibility analysis is planned, to show how more sensors, e.g. Sentinel-1, Sentinel-3, MODIS or Landsat can be added to the EO-Compass. The comparison of different sensors might also improve the expressiveness of the individual maps and allow for additional analyses, e.g. the effects of seasonal cloud cover on the different sensors or their individual performance. Although a general value of suitability estimations was shown, a major research topic remains to estimate the suitability of Sentinel-2 data for specific applications, also known as fitness-for-use.
